# Inter-observer variation in LV analysis in a dedicated CMR unit: the impact of audit and consensus guideline on reproducibility

**DOI:** 10.1186/1532-429X-16-S1-P372

**Published:** 2014-01-16

**Authors:** Richard Coulden, Emer Sonnex

**Affiliations:** 1 Dept Radiology & Diagnostic Imaging, University of Alberta Hospital, Edmonton, Alberta, Canada

## Background

Cardiac MRI (CMRI) is reported to be accurate and reproducible in the assessment of left ventricular function. This, however, is generally by experienced observers using automated or semi-automated software. Many departments still rely on manual contour tracing with trainees increasingly performing analysis but is reproducibility still good? This study assess the impact of the observer experience on LV function assessment and the role of consensus guidelines in raising standards among trainees.

## Methods

20 LV data sets of varying volumes and ejection fractions (EF) were anonymized. Each data set comprised 2 and 4 chamber long axis cine SSFPs and contiguous short axis cine SSFPs from base to apex. LV volumes (LVEDV, LVESV), end diastolic muscle mass (EDMM), and EF were manually evaluated using Argus software (Siemens Medical Solutions, Erlangen) by all those regularly analyzing data in our department (7 experienced operators (> 2 years CMR experience) and 4 inexperienced operators (< 1 year CMR experience)). Inter-observer variability for all parameters was assessed, using the mean of all expert observers as the reference. Analysis of saved contours for all observers showed a small number of common causes of variability. Based on these, consensus guidelines were agreed and instituted. 4 of the experienced and all the inexperienced observers repeated the analysis of the 10 most problematic data sets after 3 months. Inter-operator variances for analyses before and after introduction of guidelines were compared.

## Results

The department as a whole showed wide inter-observer variation for all parameters (mean standard deviation for EF, LVEDV, LVESV and EDMM were 3.8%, 10.8 mls, 10.5 mls and 23.6 gms respectively). As expected, there was greater variation between inexperienced observers than experienced observers (mean SD of variation in EF for inexperienced was 4.9% compared with 2.7% for experienced, LVEDV 12.3 mls and 7.6 mls, LVESV 11.9 mls and 7.4 mls, EDMM 29.2 gms and 16.0 gms). Following introduction of consensus guidelines, mean SD for EF fell to 2.7%, LVEDV to 7.2 mls, LVESV to 6.7 mls. There was little change in mean SD for EDMM (18.8 gms). The use of guidelines eliminated differences between experienced and inexperienced observers for all parameters.

## Conclusions

Reported reproducibility of LV function measurements by CMRI is high for experienced observers but this may not be true in large departments or when observers are inexperienced. Internal audit should be routine for validating practice and consensus guidelines can help in raising standards to meet published values.

## Funding

None.

**Figure 1:**
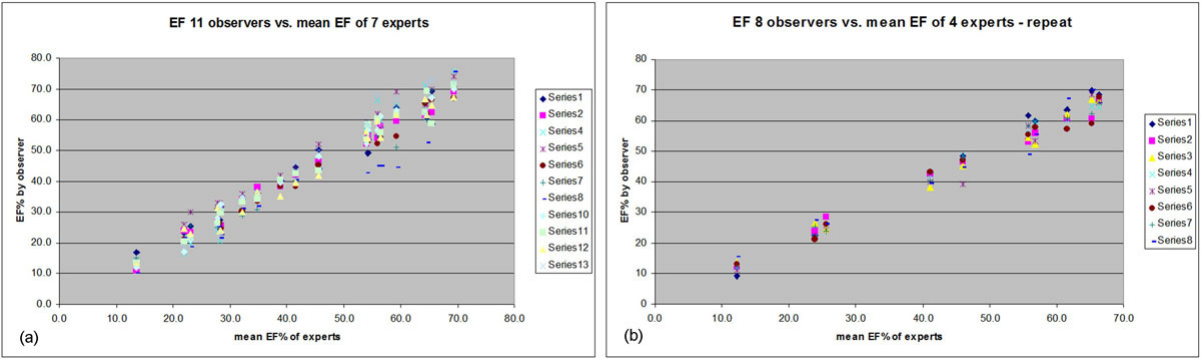
**Graphs comparing variability of EF% measurement in multiple observers compared with mean EF% of expert group before (a) and after (b) introduction of consensus rules and training**.

